# Atrioventricular plane displacement and regional function to predict outcome in pulmonary arterial hypertension

**DOI:** 10.1007/s10554-022-02616-w

**Published:** 2022-04-23

**Authors:** Anthony Lindholm, Barbro Kjellström, Felicia Seemann, Marcus Carlsson, Roger Hesselstrand, Göran Rådegran, Håkan Arheden, Ellen Ostenfeld

**Affiliations:** 1 Department of Clinical Sciences Lund, Clinical Physiology and Skåne University Hospital, Lund University, 221 85 Lund, Sweden; 2 Cardiology Unit, Department of Medicine, Karolinska Institute, Stockholm, Sweden; 3 Department of Biomedical Engineering, Faculty of Engineering, Lund University, Lund, Sweden; 4 Department of Clinical Sciences Lund, Rheumatology, and the Clinic for Rheumatology, Skåne University Hospital, Lund University, Lund, Sweden; 5 Department of Clinical Sciences Lund, Cardiology, and the Section for Heart Failure and Valvular Disease, Skåne University Hospital, Lund University, Lund, Sweden

**Keywords:** Pulmonary hypertension, Prognosis, Death, Lung transplantation, Left ventricle, Right ventricle

## Abstract

To investigate if left and right atrioventricular plane displacement (AVPD) or regional contributions to SV are prognostic for outcome in patients with pulmonary arterial hypertension (PAH). Seventy-one patients with PAH and 20 sex- and age-matched healthy controls underwent CMR. Myocardial borders and RV insertion points were defined at end diastole and end systole in cine short-axis stacks to compute biventricular volumes, lateral (SV_lat%_) and septal (SV_sept%_) contribution to stroke volume. Eight atrioventricular points were defined at end diastole and end systole in 2-, 3- and 4-chamber cine long-axis views for computation of AVPD and longitudinal contribution to stroke volume (SV_long%_). Cut-off values for survival analysis were defined as two standard deviations above or below the mean of the controls. Outcome was defined as death or lung transplantation. Median follow-up time was 3.6 [IQR 3.7] years. Patients were 57 ± 19 years (65% women) and controls 58 ± 15 years (70% women). Biventricular AVPD, SV_long%_ and ejection fraction (EF) were lower and SV_lat%_ was higher, while SV_sept%_ was lower in PAH compared with controls. In PAH, transplantation-free survival was lower below cut-off for LV-AVPD (hazard ratio [HR] = 2.1, 95%CI 1.2–3.9, p = 0.02) and RV-AVPD (HR = 9.8, 95%CI 4.6–21.1, p = 0.005). In Cox regression analysis, lower LV-AVPD and RV-AVPD inferred lower transplantation-free survival (LV: HR = 1.16, p = 0.007; RV: HR = 1.11, p = 0.01; per mm decrease). LV-SV_long%_, RV-SV_long%_, LV-SV_lat%_, RV-SV_lat%_, SV_sept%_ and LV- and RVEF did not affect outcome. Low left and right AVPD were associated with outcome in PAH, but regional contributions to stroke volume and EF were not.

## Introduction

Pulmonary arterial hypertension (PAH) is a rare disease with high morbidity and mortality rates when right heart failure is developed [1, 2].

Cardiac magnetic resonance imaging (CMR) is the gold standard for assessment of ventricular volumes and global function, especially on the right side of the heart [3]. Currently known prognostic factors for PAH with CMR are increased right ventricular (RV) volume, reduced left ventricular (LV) volume, reduced RV ejection fraction (EF) and reduced stroke volume (SV) [1]. However, these are crude measures as they do not take regional ventricular function into account and the role of RVEF as an outcome measure in PAH has been debated [4, 5]. Therefore, new methods to find better metrics of impaired cardiac function for improved risk assessment and treatment strategy planning are warranted [1].

One proposed metric is the regional deformation of the heart during contraction, and it has earlier been shown that RV longitudinal strain is prognostic in PAH [6]. While strain evaluates myocardial deformation, the atrioventricular plane displacement (AVPD) and regional contribution to SV assess the resulting changes of the ventricular structures [7, 8].

Ventricular SV is generated from longitudinal shortening and radial contraction. The longitudinal component (SV_long%_) can be quantified from AVPD and the radial component can be calculated from the septal contribution to SV (SV_sept%_) and lateral contribution to SV (SV_lat%_) measured by CMR [8].

The major contributor to SV in both the LV (60%) and the RV (80%) in healthy subjects is SV_long%_ [8]. It has been shown that patients with pulmonary hypertension of multiple etiologies have lower biventricular AVPD and LV-SV_long%_ than healthy controls [9]. However, it is unknown if AVPD or regional contributions to SV are prognostic markers for outcome in PAH. Therefore, the aim of this study was to investigate if regional longitudinal and radial markers of biventricular function have prognostic implications in patients with PAH.

## Method

### Study population

Seventy-six adult patients diagnosed with PAH who underwent CMR and right heart catheterization on clinical indication from 2003 to 2015 were included [1]. Five of these patients *were subsequently excluded* due to inadequate CMR images; *f*our lacked *2-, 3-, or 4-chamber views and one had poor image quality. This left 71 patients included for analysis.*

Outcome was defined as a composite of death or lung transplantation with end of follow-up 2019-09-01. CMR images and clinical data from 20 sex and age-matched healthy volunteers included in previous studies from our group were analyzed for definition of cut-off from normal values [10, 11]. Controls had no reported diseases or medications and had normal ECG and blood pressure.

The investigation conforms with the principles outlined in the Declaration of Helsinki. All participants gave written informed consent prior to study procedures. The study was approved by the Regional Ethical Review Board of Lund.

### CMR

#### Image acquisition

Two 1.5 Tesla scanners [Aera (Siemens, Erlangen, Germany) or Achieva (Philips, Best, the Netherlands)] were used. Standard cine balanced steady-state free precession images of three long-axis views and a short-axis stack covering the entire heart were acquired in supine position with a cardiac coil, using electrocardiography triggering during end-respiratory breath-hold. Typical image parameters for Siemens were: Temporal resolution of 46 ms reconstructed to 25 time phases per cardiac cycle, 60° flip angle, 3-ms repetition time, 1.4-ms echo time, and slice thickness 6 mm with 2-mm slice gap; and for Philips: Temporal resolution of 47 ms reconstructed to 30 time phases per cardiac cycle, 60° flip angle, 3-ms repetition time, 1.4-ms echo time and slice thickness 8 mm with no slice gap. Typical in-plane resolution was 1.5 × 1.5 mm. Late gadolinium enhancement images were acquired 15–20 min after gadolinium-based contrast agent (0.1–0.2 mmol/kg, according to clinical practice and kidney function) injection in the corresponding short- and long-axis view as for the standard cine balanced steady-state free precession images. All images were obtained in supine position at end-expiratory breath-hold. Typical image parameters: 8 mm slice thickness (no gap) with in-plane resolution 1.5 × 1.5 mm and prospective electrocardiography gating. Inversion time was chosen to null remote myocardium [12].

### Image analysis

Image analysis was performed at end diastole and end systole with the freely available software Segment v2.2 (http://segment.heiberg.se) [13]. Endocardial and epicardial contours in the short axis stack were manually delineated in RV and LV in all slices. End-diastolic volume (EDV), end-systolic volume (ESV), and EF were calculated from the endocardial borders with papillary muscles and trabeculations included in the blood pool volume [14]. Volumes were indexed to body surface area. Stroke volume was computed from EDV-ESV and EF from SV/EDV. Cardiac output was calculated from LVSV multiplied by heart rate. Myocardial mass was computed as the volume encompassed between the endocardial and epicardial contours times myocardial density (1.05 g/ml). The evaluation of late gadolinium enhancement images was performed visually (A.L. and E.O.) and categorized as absent, late gadolinium enhancement in the RV insertion points, in the septum, in the lateral LV wall or in the RV wall. Sixty of the 71 patients were analyzed for late gadolinium enhancement, with ten patients not receiving gadolinium due to impaired kidney function and with one patient having poor image quality.

The metrics AVPD, SV_long%,_ SV_lat%_ and SV_sept%_ were calculated as described previously [8, 9, 15].

**Fig. 1:**
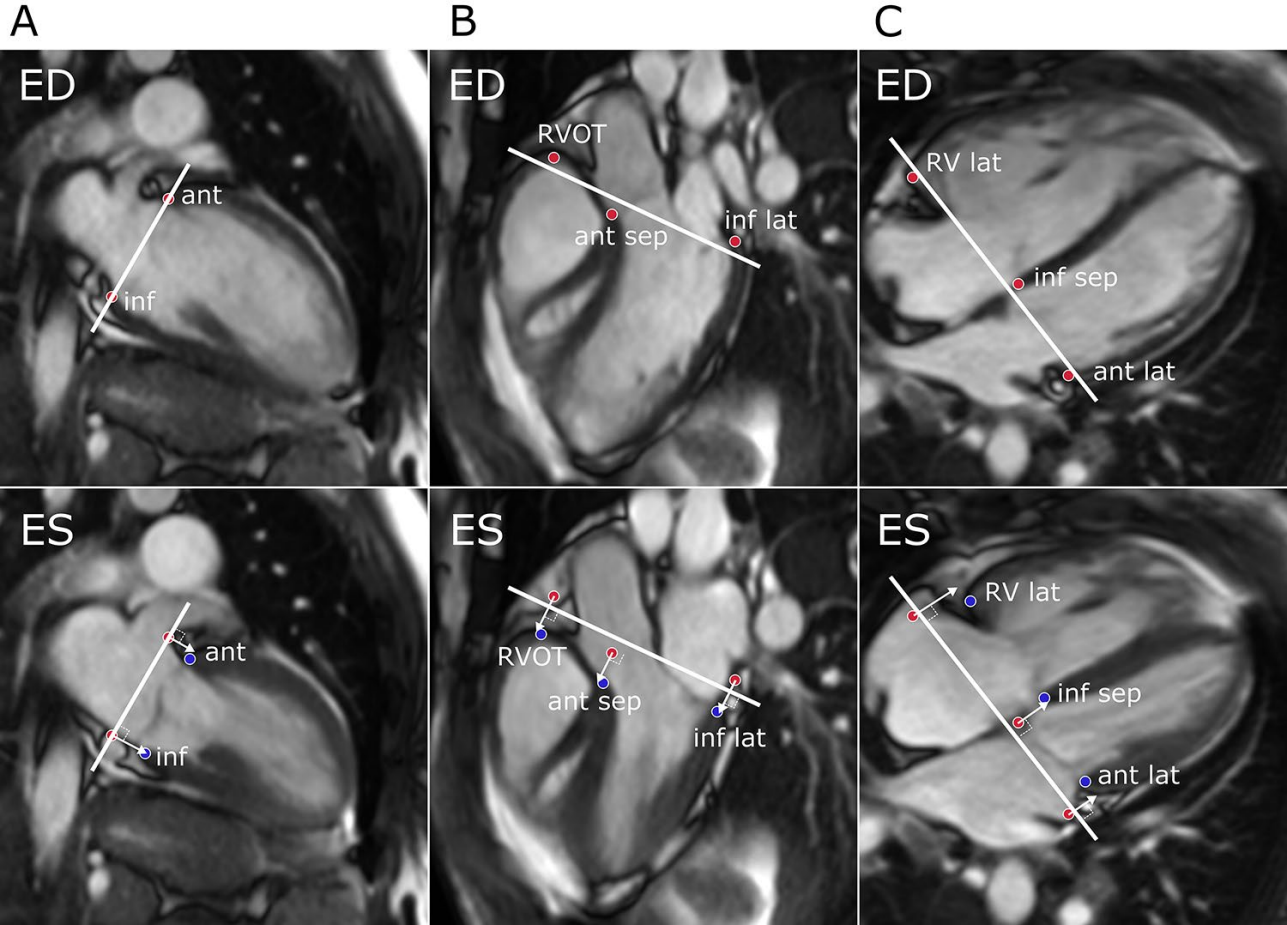
Measurement of AVPD in the LV and RV. Panel **A** demarcate the 2-chamber view, Panel **B** the 3-chamber view and panel **C** the 4-chamber view. The upper row shows end diastole (ED) and the lower row end systole (ES). Points were marked in the anterior LV wall (ant) and inferior LV wall (inf) from 2- chamber, right ventricular outflow tract (RVOT), anterior septum (ant sep) and inferior lateral LV wall (inf lat) from 3-chamber view and right ventricular lateral wall RV (RV lat), inferior septum (inf sep) and anterior lateral LV wall (ant lat) from 4chamber view. Red points demarcate the points in ED and blue points in ES. The end-diastolic points are interpolated to the end systolic images. The atrioventricular plane was defined as the line crossing the points in ED, marked with white lines. AVPD was calculated as the mean perpendicular longitudinal displacement for the points between ED and ES marked with white arrows. (Color figure online)

For AVPD analysis, eight points in the base of the ventricles were manually annotated at end diastole and end systole, assisted by a validated semi-automated algorithm [16]. Two points were marked in two-chamber view, anterior LV wall (ant) and inferior LV wall (inf), three points were marked in three-chamber view in the RV outflow tract (RVOT), anterior septum (ant sep) and inferior lateral LV wall (inf lat) and finally three points were marked in four-chamber view in lateral RV wall (RV lat), inferior septum (inf sep) and anterior lateral LV wall (ant lat) (Fig. 1).

The atrioventricular plane was defined as the line crossing these points in end diastole, and LV-AVPD was calculated as the mean longitudinal displacement, perpendicular to the atrioventricular plane, from ED to ES (∆) in the six LV points (Fig. 1) according to previously described [8].

1 $$\small \mathrm{LV}-\mathrm{AVPD}= \frac{\Delta \mathrm{ant}+\Delta \mathrm{ant lat}+\Delta \mathrm{inflat}+\Delta \mathrm{inf}+\Delta \mathrm{infsep}+\Delta \mathrm{ant sep}}{6}$$

Likewise, RV-AVPD was calculated as the mean longitudinal displacement, perpendicular to the atrioventricular plane, from ED to ES (∆) using the four RV points (Fig. 1) according to previously described [8].

2 $$\small \mathrm{RV}-\mathrm{AVPD}= \frac{\frac{\Delta \mathrm{ant sep}+\Delta \mathrm{infsep}}{2}+\Delta \mathrm{RVOT}+\Delta \mathrm{RV lat}}{3}$$

The CMR surrogate of tricuspid annular plane systolic excursion (CMR-TAPSE) was assessed in the 4-chamber view and calculated as the displacement of the RV lat-point.

**Fig. 2:**
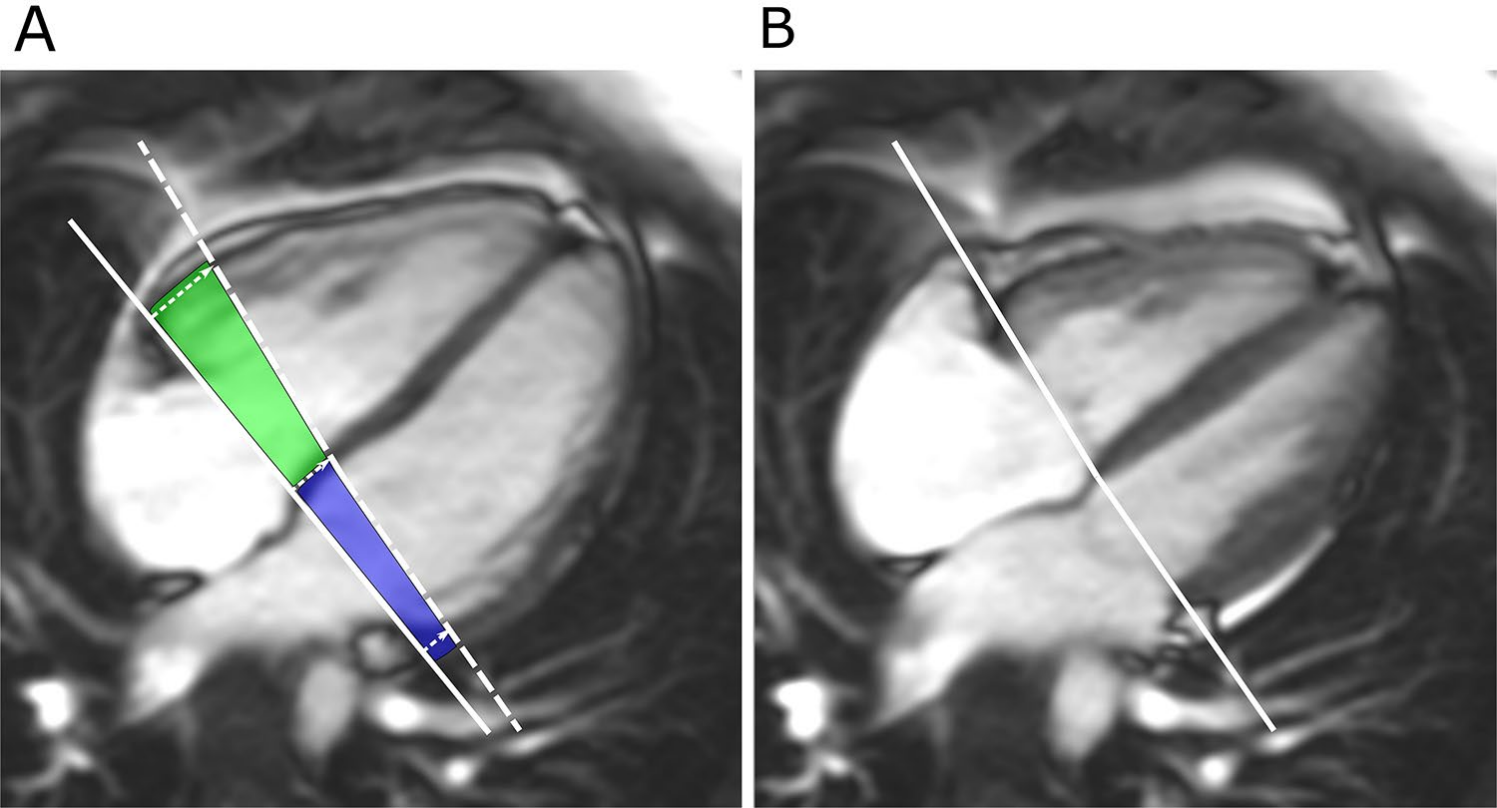
Visualization of longitudinal contribution to stroke volume. Cardiac magnetic resonance images in four-chamber view. **A** Solid lines represent the atrioventricular (AV) plane at end diastole, and **B** dashed lines represent the atrioventricular plane at end systole. The end-systolic line is interpolated to the end-diastolic image. The AV-plane displacement is the distance between the solid line in end diastole and the dashed line in end systole as shown to the left. The longitudinal contribution to stroke volume is computed as the volume encompassed by the AV-plane displacement, here illustrated with blue color in the left ventricle and green color in the right ventricle. (Color figure online)

The SV_long%_ for each ventricle was calculated as AVPD of that ventricle multiplied by the mean short-axis epicardial areas from the two largest basal ventricular slices divided by the SV (Fig. 2) [17].

**Fig. 3:**
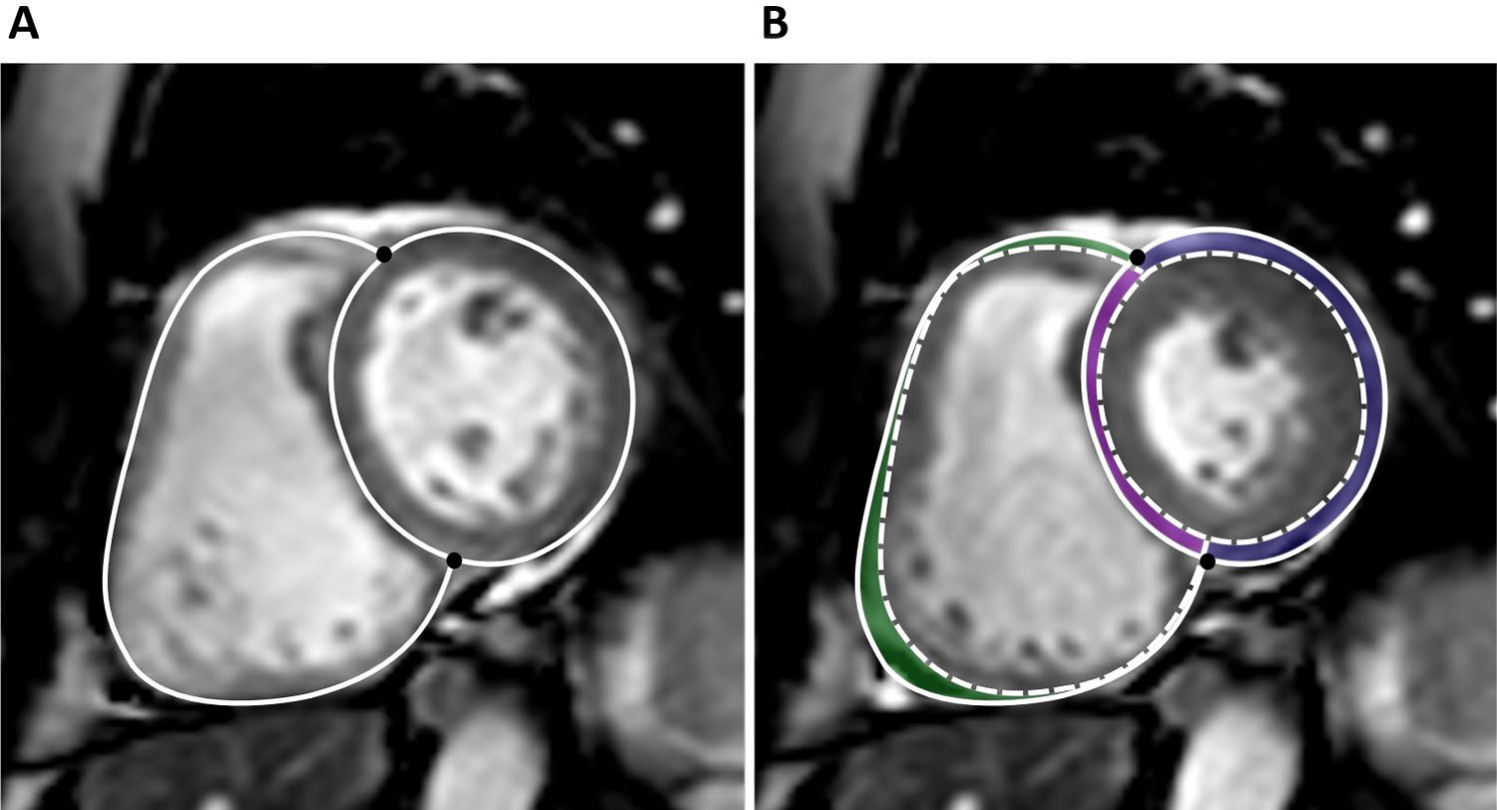
CMR images in short axis view. **A** Solid lines represent the epicardial borders and black circles RV insertions points at end diastole, and **B** dashed lines represent the epicardial borders at end systole. The end diastolic contour is interpolated to the end-systolic image. The area between the solid and dashed lines inside the RV insertion points (purple area, panel **B**) represents the septal contribution to stroke volume*,* which was summed from all short-axis slices. The area between the solid and dashed lines outside of the RV insertion points represent the lateral contribution to stroke volume*,* which was summed from all short-axis slices (green area, lateral contribution to RV, blue area lateral contribution to left ventricle). (Color figure online)

The SV_lat%_ and SV_sept%_ were calculated from the delineated epicardial contours of the free LV and RV walls as well as the septal wall. The RV insertion points were used to define the extent of the septum (Fig. 3A). Due to longitudinal shortening of the ventricles the basal slices only have the septum in diastole, and therefore only slices with ventricular septum present in both end diastole and end systole were used for calculation of lateral and septal contributions to SV. The lateral SV was calculated as the area between the epicardial border at end diastole and end systole multiplied by slice thickness, including slice gap, summed for all included slices. The SV_lat%_ was computed by dividing this volume by the total SV for the respective ventricle, generating LV-SV_lat%_ and RV-SV_lat%_ (Fig. 3). The septal SV was calculated as the area between the septal border at end diastole and end systole multiplied by slice thickness, for all included slices (Fig. 3). The SV_sept%_ was defined as septal SV divided by LV-SV. Positive SV_sept%_ was defined as septal contribution to LV-SV and negative as contribution to RV-SV.

### Right heart catheterization

Right heart catheterization was performed on clinical indication in supine position with local anesthesia using a triple-lumen Swan-Ganz catheter. No healthy controls had right heart catheterization. Pulmonary arterial pressure, right atrial pressure, and pulmonary artery wedge pressure were measured as an average over several heart beats and during free breathing. Pulmonary vascular resistance was defined as mean pulmonary arterial pressure minus pulmonary artery wedge pressure, divided by cardiac output. Cardiac output was measured from thermodilution according to clinical protocol [18]. Non-invasive systemic pressures were acquired with brachial cuff and sphygmomanometer.

### Statistical analysis

Statistical analysis was performed in IBM SPSS Statistics for Windows, Version 25.0 (IBM Corp, Armonk, NY, USA) and GraphPad Prism version 7.00 for Windows (GraphPad Software, La Jolla California USA, www.graphpad.com). Continuous variables were expressed as mean ± standard deviation (SD) and 95% confidence interval, or median and interquartile range [IQR] according to normal distribution. Normal distribution was assessed using Shapiro–Wilk tests. Categorical variables were expressed as absolute numbers and proportion (in percentage). Group comparisons for parametric data were analyzed with independent samples T-tests, and Mann–Whitney U-test for non-parametric data. Two-sided Chi-square was used for nominal data. Values were predefined as being *altered* when more than 2SD from the mean of the control group and being *within limits* of normal distribution when within 2SD. Kaplan–Meier plots were used for survival curves with Cox regression analysis for calculating hazard ratio (HR) and log-rank test for p-values. Cox regression analysis was performed as univariate analysis for continuous variables. In the bivariate analysis, variables with p < 0.1 from the univariate analysis were included. A two-sided p-value < 0.05 was considered statistically significant.

## Results

Seventy-one patients with PAH (57 ± 19 years, 46 women) were included (Table 1). Thirty-two were diagnosed with idiopathic/familial PAH and 39 with PAH associated with connective tissue disease (25 of which owing to systemic sclerosis). Median follow-up time from CMR to an event or study completion was 3.6 [3.7] years. Among patients, 69% were scanned in conjunction with diagnosis. During CMR examination 82% were receiving PAH specific medical treatment (Table 1).

**Table 1:** Characteristics for healthy controls compared with patients with PAH, and patients with no death or lung transplantation compared with patients with death or lung transplantation

	Control (n = 20)	PAH (n = 71)	p-value control/PAH	No death or tx (n = 29)	Death or tx (n = 42)	p-value no death or tx/death or tx
Biometrics						
Age (years)	58 ± 15	57 ± 19	0.9	50 ± 21	62 ± 17	**0.008**
Sex; women (%)	70	65	0.7	76	57	0.1
BSA (m^2^)	1.8 ± 0.2	1.8 ± 0.2	0.5	1.8 ± 0.2	1.9 ± 0.2	0.5
NIBP, systolic (mmHg)	127 ± 12	124 ± 19^a^	0.4	128 ± 17	121 ± 20^a^	0.1
NIBP, diastolic (mmHg)	76 ± 10	77 ± 12^a^	0.5	79 ± 13	77 ± 12^a^	0.5
NT-proBNP (ng/L)	–	1978 [3500]^c^		1221 [2545]^d^	2325 [4357]^e^	0.08
Smoker; yes/ex (%)	0/0	4/38		0/35	7/41	0.3
Incident/prevalent (%)	–	69/31		83/17	60/40	**0.04**
Right heart catheterization^a^						
sPAP (mmHg)	–	73 ± 18^a^		73 ± 17	74 ± 19^a^	0.8
mPAP (mmHg)	–	47 ± 13^a^		48 ± 14	45 ± 12^a^	0.3
dPAP (mmHg)	–	28 ± 10^a^		28 ± 9	28 ± 10^a^	1.0
PAWP (mmHg)	–	8 ± 4^b^		8 ± 4	7 ± 4^b^	0.4
mRAP (mmHg)	–	7 ± 6^a^		6 ± 6	8 ± 6^a^	0.3
PVR (wood units)	–	9 ± 5^a^		9 ± 4	9 ± 6^a^	0.5
Comorbidities						
Diabetes (%)	0	21		21	21	0.9
Raynaud’s (%)	0	35		31	38	0.5
Ischemic heart disease (%)	0	10		3	14	0.1
Medical treatment at CMR						
PAH treatment (%)		82		83	81	0.8
Single therapy (%)		44		41	45	0.7
Dual therapy (%)		35		38	33	0.7
Triple therapy (%)		3		3	2	0.8

Data are presented as mean ± standard deviation, median [interquartile range] or proportion in percentage

*Death or tx* death or lung transplantation, *PAH* pulmonary arterial hypertension, *BSA* body surface area, *NIBP* non‐invasive systemic blood pressure, *NT-proBNP* N-terminal pro b-type natriuretic peptide, *Incident/prevalent* patients with cardiac magnetic resonance imaging (CMR) at time of diagnosis/with known diagnosis, *sPAP* systolic pulmonary artery pressure, *mPAP* mean pulmonary artery pressure, *dPAP* diastolic pulmonary artery pressure, *PAWP* pulmonary artery wedge pressure, *mRAP* mean right atrial pressure, *PVR* pulmonary vascular resistance, *Single therapy* treated with one PAH medication (endothelin receptor antagonist, phosphodiesterase type 5 inhibitor or prostanoid), *dual therapy* treated with two PAH medications, *triple therapy* treated with three PAH medications. Significant values in bold

^a^n-1 (data missing for one subject)

^b^n-2 (data missing for two subjects)

^c^n = 59

^d^n = 24

^e^n = 35

Patients had lower EF, SV, AVPD and SV_long%_ and higher SV_lat%_ in both ventricles than controls (Tables 2, 3). LV-EDV and SV_sept%_ were lower and RV-EDV was higher in patients than controls (Tables 2, 3).

**Table 2:** Cardiac magnetic resonance (CMR) data for healthy controls compared with patients with PAH, and patients with no death or lung transplantation compared with patients with death or lung transplantation

	Control (n = 20)	PAH (n = 71)	p-value control/PAH	No death or tx (n = 29)	Death or tx (n = 42)	p-value No death or tx/death or tx
Time diagnosis to CMR (days)	–	2 [112]^a^		2 [63]^b^	4 [132]	0.7
Resting heart rate (bpm)	61 ± 10	79 ± 14	** < 0.0001**	74 ± 15	82 ± 13	**0.03**
*Left ventricle*						
LVEF (%)	61 ± 4	53 ± 9	** < 0.0001**	53 ± 8	53 ± 9	0.9
LV-EDV (ml)	166 ± 32	127 ± 34	** < 0.0001**	133 ± 37	123 ± 32	0.2
LV-ESV (ml)	65 ± 15	59 ± 19	0.2	63 ± 20	57 ± 18	0.2
LV-SV (ml)	101 ± 21	68 ± 21	** < 0.0001**	71 ± 22	66 ± 21	0.3
LV-SVI (ml/m^2^)	56 ± 8	37 ± 11	** < 0.0001**	39 ± 10	35 ± 11	0.2
LVMI (g/m^2^)	45 ± 11	50 ± 17	0.3	49 ± 18	50 ± 17	1.0
Cardiac output (L/min)	6.0 ± 1.4	5.2 ± 1.6	**0.04**	5.1 ± 1.5	5.3 ± 1.7	0.6
*Right ventricle*						
RVEF (%)	56 ± 4	35 ± 11	** < 0.0001**	36 ± 11	34 ± 11	0.3
RV-EDV (ml)	183 ± 42	238 ± 76	** < 0.0001**	229 ± 58	245 ± 87	0.4
RV-ESV (ml)	80 ± 21	161 ± 70	** < 0.0001**	150 ± 54	168 ± 79	0.3
RV-SV (ml)	103 ± 24	78 ± 21	** < 0.0001**	80 ± 23	76 ± 20	0.5
RV-SVI (ml/m^2^)	57 ± 10	42 ± 11	** < 0.0001**	44 ± 12	41 ± 10	0.3
RVMI (g/m^2^)	12 ± 3	23 ± 8	** < 0.0001**	23 ± 8	23 ± 9	1.0

Data are presented as mean ± standard deviation or median [interquartile range]

*Death or tx* death or lung transplantation, *PAH* pulmonary arterial hypertension, *CMR* cardiac magnetic resonance imaging, *LV* left ventricle, *EF* ejection fraction, *EDV* end‐diastolic volume, *ESV* end‐systolic volume, *SV* stroke volume, *SVI* stroke volume index, *MI* myocardial mass index, *RV* right ventricle. Significant values in bold

^a^n = 70

^b^n = 28

**Table 3:** AVPD and regional contributions to stroke volume for healthy controls compared with patients with PAH, and patients with no death or lung transplantation compared with patients with death or lung transplantation

	Control (n = 20)	PAH (n = 71)	p-value control/PAH	No death or tx (n = 29)	Death or tx (n = 42)	p-value no death or tx/death or tx
*Left ventricle*						
LV-AVPD (mm)	16 ± 2	11 ± 3	** < 0.0001**	12 ± 3	10 ± 3	**0.02**
LV-SV_long%_ (%)	57 ± 8	51 ± 11	**0.02**	53 ± 11	49 ± 11	0.2
LV-SV_lat%_ (%)	29 ± 9	48 ± 17	** < 0.0001**	45 ± 12	51 ± 19	0.1
SV_sept%_ (%)	9 ± 4	4 ± 14	**0.01**	5 ± 12	4 ± 15	0.6
*Right ventricle*						
RV-AVPD (mm)	22 ± 3	12 ± 4	** < 0.0001**	13 ± 4	11 ± 3	**0.02**
RV-SV_long%_ (%)	85 ± 11	72 ± 19	** < 0.0001**	75 ± 20	70 ± 19	0.3
RV-SV_lat%_ (%)	27 ± 9	34 ± 16	**0.01**	31 ± 14	36 ± 17	0.1

Data are presented as mean ± standard deviation

*Death or tx* death or lung transplantation, *PAH* pulmonary arterial hypertension, *LV* left ventricle, *AVPD* atrioventricular plane displacement, *SV*_*lat%*_ lateral contribution to stroke volume, *SV*_*long%*_ longitudinal contribution to stroke volume, *SV*_*sept%*_ septal contribution to stroke volume, *RV* right ventricle. Significant values in bold

There was no late gadolinium enhancement in four patients (7%) and late gadolinium enhancement was present in 56 patients (93%). Of those, late gadolinium enhancement was present at the RV insertion points in all 56 patients, in the septum in two patients, in the lateral LV wall in five patients and in the RV in one patient. There was no significant difference in patients without events compared to patients with events with regards to late gadolinium enhancement (p = 0.5). There was no difference in LV-AVPD or RV-AVPD between patients with late gadolinium enhancement compared to patients without (LV: p = 0.2, RV: p = 0.3).

**Fig. 4:**
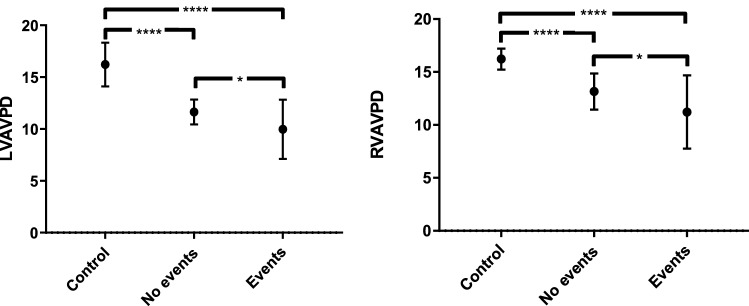
Atrioventricular plane displacement (AVPD) in controls, patients without events and patients with events in mean ± CI (95%). Left panel shows LV AVPD. Right panel shows RV AVPD. Control group (n = 20), patients without events (n = 29) and patients with events (n = 42). *p < 0.05, ****p < 0.0001

There were 42 events, of which 36 were deaths and 6 lung transplantations (Table 1). Patients with events were older and had lower LV-AVPD and RV-AVPD than patients without events (Tables 1, 3 and Fig. 4). There was no difference related to sex or in measures obtained during right heart catheterization between patients with and without events (Table 1).

The cut-off values for survival analysis were, for LV-AVPD: < 12.0 mm, RV-AVPD: < 16.8 mm, LVSV_long%_: < 41.4%, RVSV_long%_: < 63.8%, SV_sept%_: < 0.8%, LVEF: < 53% and RVEF: < 48% as well as LVSV_lat%_: > 46.9% and RVSV_lat%_: > 44.5%. The mean and SD values are presented in Tables 2, 3.

**Fig. 5:**
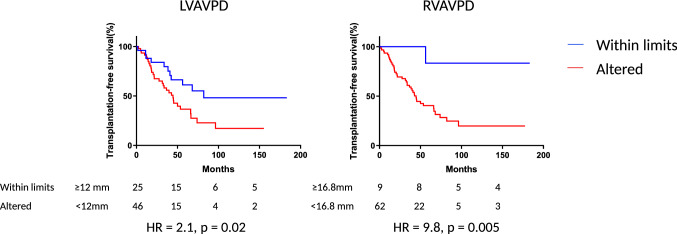
Kaplan–Meyer survival analysis of transplantation free survival regarding left ventricular atrio-ventricular plane displacement (LV-AVPD) and right ventricular atrio-ventricular plane displacement (RV-AVPD). The blue lines represent patients with values within limits and the red line patients with altered values. (Color figure online)

**Fig. 6:**
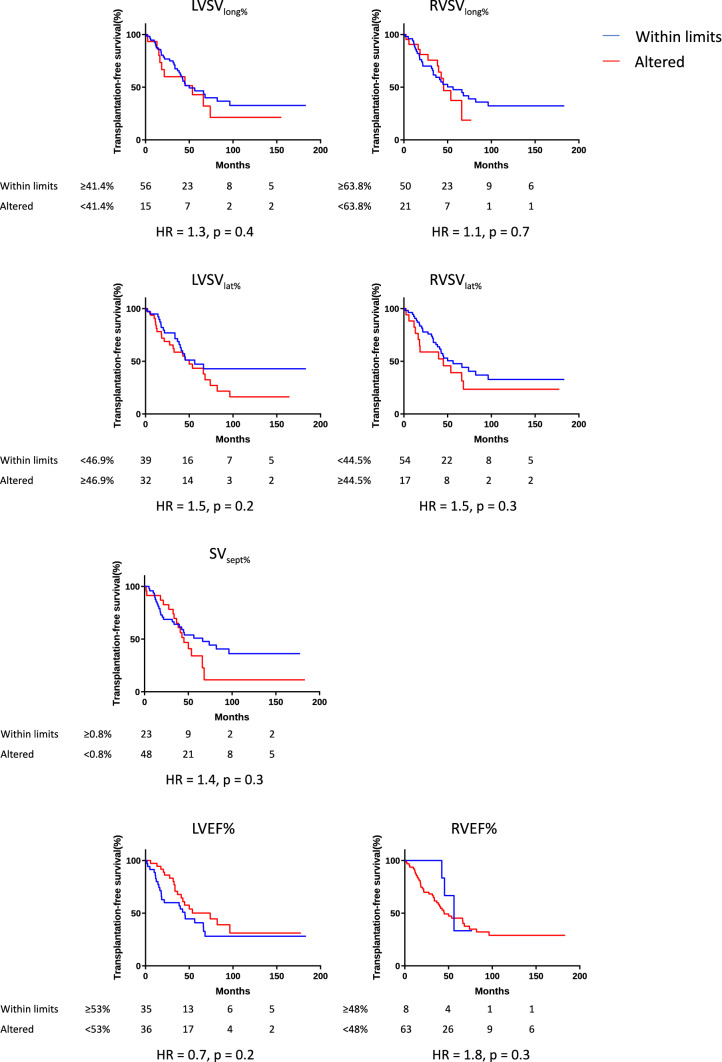
Kaplan–Meyer survival analysis of transplantation free survival regarding left ventricular longitudinal contribution to stroke volume (LV-SV_long%_), right ventricular longitudinal contribution to stroke volume (RV-SV_long%_), left ventricular lateral contribution to stroke volume (LV-SV_lat%_), right ventricular contribution to stroke volume (RV-SV_lat%_), septal contribution to stroke volume (SV_sept%_), left ventricular ejection fraction (LVEF%) and right ventricular ejection fraction (RVEF%). The blue lines represent patients with values within limits and the red line patients with altered values. (Color figure online)

Kaplan–Meier analysis showed lower transplantation-free survival in patients with LV-AVPD and RV-AVPD 2SD below normal values compared with patients with values within limits (Fig. 5). Patients with low values of LV-SV_long%_, RV-SV_long%_, SV_sept%_, LVEF or RVEF or high values of LV-SV_lat%_ or RV-SV_lat%_ did not have a different outcome compared with patients with values within limits (Fig. 6).

Low LV-AVPD and RV-AVPD were associated with decreased transplantation-free survival in the unadjusted analysis and remained associated with outcome when adjusting for age and for incident/prevalent patients (Table 4). CMR-TAPSE was not associated with decreased transplantation-free survival in univariate analysis but were significantly associated with prognosis in multivariate analysis adjusting for age and incident/prevalent patients (Table 4).

**Table 4:** Univariate, bivariate (adjusted for age) and multivariate (adjusted for age and prevalent/incident). Cox regression analysis for major adverse cardiac event

	Univariate HR (95% CI)	Univariate p-value	Bivariate^a^ HR (95% CI)	Bivariate^a^ p-value	Multivariate^b^ (95% CI)	Multivariate^b^ p-value
LV-AVPD	1.16 (1.04–1.28)	**0.007**	1.16 (1.04–1.29)	**0.008**	**1.16 (1.04–1.29)**	**0.008**
RV-AVPD	1.11 (1.03–1.20)	**0.01**	1.12 (1.03–1.21)	**0.01**	**1.12 (1.03–1.22)**	**0.01**
SV_sept%_	1.00 (0.98–1.02)	1.0	–	–	–	–
LV-SV_lat%_	1.01 (0.99–1.03)	0.3	–	–	–	–
RV-SV_lat%_	0.98 (0.96–1.00)	0.09	0.98 (0.96–1.00)	0.06	0.98 (0.96–1.01)	0.1
LV-SV_long%_	1.02 (1.00–1.05)	0.08	1.02 (1.00–1.05)	0.09	1.02 (0.99–1.05)	0.2
RV-SV_long%_	1.01 (1.00–1.03)	0.1	1.01 (1.00–1.03)	0.2	1.01 (0.99–1.03)	0.2
LVEF%	1.00 (0.96–1.03)	0.9	–	–	–	–
RVEF%	1.01 (0.98–1.04)	0.4	–	–	–	–
RVSV	1.00 (0.99–1.02)	0.7	–	–	–	–
LVEDV	1.01 (1.00–1.02)	0.1	1.00 (0.99–1.01)	0.5	1.00 (0.99–1.01)	0.8
RVEDV	1.00 (0.99–1.00)	0.4	–	–	–	–
TAPSE	1.06 (1.00–1.13)	0.05	1.06 (1.00–1.14)	0.07	1.08 (1.01–1.16)	**0.046**
Incident/prevalent	0.54 (0.29–1.00)	0.05	0.53 (0.29–0.99)	**0.045**	–	–
Age	1.03 (1.01–1.05)	**0.003**	–	–	–	–

Univariate analysis for increased risk of lung transplantation or death with continuous variables

*HR* hazard ratio for decrease in each incremental step, *CI* confidence interval, *LV-AVPD* left ventricular atrio-ventricular displacement, *RV-AVPD* right ventricular atrio-ventricular displacement, *SV*_*sept%*_ septal contribution to stroke volume, *LV-SV*_*long%*_ left ventricular longitudinal contribution to stroke volume, *RV-SV*_*long%*_ right ventricular longitudinal contribution to stroke volume, *LV-SV*_*lat%*_ left ventricular lateral contribution to stroke volume, *RV-SV*_*lat%*_ right ventricular lateral contribution to stroke volume, *LVEF* left ventricular ejection fraction, *RVEF* right ventricular ejection fraction, *RVSV* right ventricular stroke volume, *LVEDV* left ventricular end diastolic volume, *RVEDV* right ventricular end diastolic volume, *TAPSE* tricuspid annular systolic excursion measured with cardiac magnetic resonance, *Incident/prevalent* patients with cardiac magnetic resonance at time of diagnosis/with known diagnosis. Significant values in bold

^a^Bivariate analysis adjusted for age

^b^Multivariate analysis adjusted for age and incident/prevalent

## Discussion

The main finding of this study was that low AVPD in both ventricles were associated with increased risk of lung transplantation and death in patients with PAH. The risk was related to the magnitude of AVPD. Of note, LVEF and RVEF were not different between patients with and without events but AVPD for both ventricles *were* different. Thus, we found that prognosis in this PAH patient cohort was related to longitudinal but not global ventricular function. The association between LV longitudinal function and survival has to our knowledge not been shown before.

### Survival analysis

We found that each mm decrease in LV-AVPD and RV-AVPD was associated with decreased transplantation-free survival of 16% and 11%, respectively. Although there is a lack of previous studies analyzing AVPD, the findings for RV-AVPD is in line with the earlier meta-analysis by Baggen et al*.* on tricuspid annular plane systolic excursion (TAPSE) in PAH that found a HR of mortality being 1.72 per 5 mm decrease in TAPSE (p < 0.001) [19].

When applying Kaplan Meier analysis with dichotomized cut-off values below 2SD from the means of the healthy controls, we found HR > 2 for lung transplantation or death when LV-AVPD was < 12.0 mm and HR ~ 10 when RV-AVPD was < 16.8 mm. The low transplantation-free survival occurring with low RV-AVPD is in line with an echocardiographic study by Schuuring et al*.* showing that TAPSE ≤ 15 mm predicted higher mortality with a HR of 3.5 compared with TAPSE > 15 mm [20].

However, TAPSE with echocardiography has shown conflicting results, as a study by Cho et al*.* found no significant differences in mortality in patients with low TAPSE ≤ 14 mm compared with TAPSE > 14 mm [21]. In the study by Schuuring et al*.* the cut-off was computed from normal values while in the study by Cho et al*.* infra- and supra-median values were used as cut-offs [20, 21]. As dichotomized variables from normal values are more generalizable than using infra- and supra-median values from patients, we chose to use normal values. Our methodology and results are hence more aligned with the study by Schuuring et al*.* than with Cho et al*.*[20, 21].

It should be noted that although both AVPD and TAPSE measure the movement of the tricuspid annulus, the methods are different. TAPSE measures the movement of the lateral tricuspid annulus towards the apex and AVPD the movement of the entire atrioventricular plane towards the apex. This difference results in a lower AVPD value compared with TAPSE, as the highest AVPD movement often is found in the lateral tricuspid annulus and the mean AVPD includes the septal and RV outflow tract parts of AVPD. Furthermore, echocardiography and CMR AVPD-based values seem to be interchangeable in a healthy adult population [22], but less so in patients with pulmonary hypertension [23]. After adjustment for age and incident/prevalent patients, CMR-TAPSE was associated with decreased transplantation-free survival in the present study, however, this association was stronger with LV-AVPD or RV-AVPD.

A novel finding in the present study was low LV-AVPD being predictive of reduced survival. The implications of reduced LV-AVPD on outcome have not been shown previously. The cause of the failing LV could be a result of underfilling of the LV or interventricular interdependence of the heart [24]. Thus, we hypothesize that the decreased LV function seen in PAH is a sign that the RV is failing and no longer able to provide blood to the LV. Whether or not the failing LV plays a part in PAH mortality needs to be evaluated in future studies.

It should be noted that while LV-AVPD had a higher hazard ratio compared with RV-AVPD using continuous variables, RV-AVPD had a higher hazard ratio using dichotomized variables from normal values. This might suggest that the association with decreased survival due to low RV-AVPD might be more threshold dependent while the association with LV-AVPD is of a more continuous nature.

Another method of evaluating longitudinal function is cardiac deformation using longitudinal strain. It has been shown that reduced RV longitudinal strain is associated with increased risk for death, lung transplantation and functional class deterioration in pulmonary hypertension [6]. In addition, it has been shown that both LV and RV longitudinal strain improves after treatment [25]. Furthermore, intra- and interventricular dyssynchrony in pulmonary hypertension can be assessed by strain [26], while our method of regional contribution to stroke volume cannot take this into account. However, in comparison to longitudinal strain, AVPD is a method readily available by marking points at end diastole and end systole, making it easily suited for clinical application.

There were no associations between LV-SV_long%_, RV-SV_long%_, LV-SV_lat%_, RV-SV_lat%_ or SV_sept%_ and outcome. It might seem counterintuitive that there were correlations between low AVPD in both ventricles and decreased survival but not between low SV_long%_ and survival. However, low AVPD does not directly imply low SV_long%_, since SV_long%_ depends on both AVPD and the area in the short axis. So, even if AVPD decreases, SV_long%_ can be preserved or even increased if the short-axis area increases. A similar effect has been observed in patients with left heart failure with dilated LV and low SV, where LV-SV_long%_ can be preserved even with low LV-AVPD owing to larger LV basal area [17].

Neither RVEF nor LVEF were associated with decreased survival among patients with PAH in the present study. Previous studies on RVEF and survival in PAH show conflicting results [27–29]. One meta-analysis found RVEF as the strongest predictor of mortality [27]. A later meta-analysis by Dong et al*.* confirmed that RVEF was a strong predictor of all-cause death in pulmonary hypertension of several etiologies, but in patients with PAH without congenital heart disease, RVEF was, in fact, not a predictor of outcome [4, 5]. Furthermore, recent studies could not reproduce RVEF as being associated with outcome [28, 29]. Regarding the left side, previous studies have shown that the longitudinal motion has a strong prognostic significance in patients with left heart failure, independent of LVEF and age [30], and our results suggest that longitudinal pumping with AVPD might also be a better method to predict outcome than EF in PAH.

Likewise, although patients in the present study had lower SV, lower LV end-diastolic volume and higher RV end-diastolic volume compared to controls, there was no association between these three variables and survival. This is in contrast with a previous study where the same variables predicted a worse prognosis [31]. However, the latter study population included only incident patients with idiopathic PAH while the present study had a less homogenous population and included both incident and prevalent patients.

In the present study, the proportion of prevalent patients were higher in the group having events and in cox regression prevalent patients had worse prognosis when adjusted for age. This is in contrast to a study with 674 patients with idiopathic, familial and anorexigen-associated PAH, in which incident patients had worse prognosis than prevalent [32]. However, neither age nor incident/prevalent affected LV- and RV-AVPD as independent markers of outcome.

### Physiological changes in pumping

In the present study, patients with PAH had low AVPD, SV_long%_ and high SV_lat%_ in both ventricles as well as a low SV_sept%_ compared with healthy controls. This indicates an alteration in regional cardiac pumping with decreased longitudinal pumping and increased radial pumping, in both ventricles in patients with PAH.

In the present study, patients with PAH had longitudinal and lateral contributions to SV in the RV, similar to what is normally seen in the LV [8]. It has earlier been shown that longitudinal pumping is more energy-efficient than radial pumping [33]. However, in the present study RV longitudinal pumping was lower and radial pumping higher, meaning that they are moving towards a less energy-efficient state. One could hypothesize that radial pumping is better suited to pumping towards high pressures than longitudinal pumping. In PAH, the RV pressures can reach levels normally seen in the LV and hence the shift in pumping pattern could be an adaptation to increased pressure. It is known that the RV undergoes remodeling with hypertrophy in patients with PAH and it has been shown in pigs that there is a redistribution of muscle fibers with an increase in circular fibers in the pressure-loaded RV [34, 35]. It has also been shown with echocardiography that longitudinal shortening is low in patients with PAH and that improved RV function following therapy occurs solely from improved longitudinal shortening [36].

On the other hand, the decrease in longitudinal pumping and increase in radial pumping of the LV is less intuitive. We suggest that the changes in LV function could be due to diastolic and systolic ventricular interdependence of the heart or underfilling of the LV due to inadequate RV forward pumping [24, 37]. This is supported in this study by larger RV-EDV and smaller LV-EDV in patients than controls.

In PAH, the morphology of the septum changes as an effect of a change in the pressure gradient between the LV and the RV, and as the pressures equalize the septum flattens [38]. In the present study, septal movement was impaired with lower SV_sept%_ in patients compared with controls. This might be a sign of increased paradoxical septal motion, which has previously been described in patients with pressure-loaded RV and has been associated with poor outcome in precapillary pulmonary hypertension [39].

### Limitations

The study is retrospective and hence there is a risk of selection bias. However, as all patients were referred from a tertiary PAH center, where a standardized clinical routine include CMR assessment, there is a low likelihood for substantial referral bias. In addition, risks for selection bias have been minimized by screening all patients that underwent CMR during the selected time period for study participation.

In our population, both newly diagnosed and prevalent patients were included. However, we intended to investigate if AVPD and regional contribution was indicative of outcome in any patient with PAH irrespective of stage of disease progress.

Some of the comparisons, mainly RVEF and RV-AVPD, had large differences in group sizes. This was due to the method of using cut-off values from healthy controls ± 2SD for Kaplan–Meier analysis. However, the Kaplan–Meier analyses were largely in agreement with the Cox regression analysis.

The proportion of women was higher among patients without events than patients with events. It has been suggested that men have worse prognosis compared with women with PAH, although this might be owing to lower RVEF rather than sex-differences in itself [40]. However, in our study, there were no sex differences in any of the studied variables, and thus this could not explain the lower event rate in women. An earlier study from the Swedish PAH Registry has shown that when adjusting for age there was no difference in mortality between women and men in the Swedish PAH population [41].

## Conclusions

Low left and right AVPD were both associated with outcome in PAH, but regional contributions to stroke volume and EF were not. This implies that AVPD measured with CMR could be a useful tool in assessing the disease progress and risk assessment in PAH.

## Abbreviations

AVPD: Atrioventricular plane displacement
CMR: Cardiac magnetic resonance imaging
EF: Ejection fraction
HR: Hazard ratio
LV: Left ventricle
mPAP: Mean pulmonary arterial pressure
NT-proBNP: N-terminal pro b-type natriuretic peptide
PAH: Pulmonary arterial hypertension
RV: Right ventricle
SD: Standard deviation
SV: Stroke volume
SV_lat__%_: Lateral contribution to stroke volume
SV_long__%_: Longitudinal contribution to stroke volume
SV_sept__%_: Septal contribution to stroke volume
TAPSE: Tricuspid annular plane systolic excursion

## References

[1] Galiè N, Humbert M, Vachiery J-L (2016). 2015 ESC/ERS guidelines for the diagnosis and treatment of pulmonary hypertension. Eur Heart J.

[2] Kylhammar D, Persson L, Hesselstrand R, Rådegran G (2014). Prognosis and response to first-line single and combination therapy in pulmonary arterial hypertension. Scand Cardiovasc J.

[3] Benza R, Biederman R, Murali S, Gupta H (2008). Role of cardiac magnetic resonance imaging in the management of patients with pulmonary arterial hypertension. J Am Coll Cardiol.

[4] Dong Y, Pan Z, Wang D (2020). Prognostic value of cardiac magnetic resonance-derived right ventricular remodeling parameters in pulmonary hypertension: a systematic review and meta-analysis. Circ Cardiovasc Imaging.

[5] Ostenfeld E, Kjellström B (2020). The conundrum of right ventricular remodeling and outcome in pulmonary hypertension. Circ Cardiovasc Imaging.

[6] de Siqueira MEM, Pozo E, Fernandes VR (2016). Characterization and clinical significance of right ventricular mechanics in pulmonary hypertension evaluated with cardiovascular magnetic resonance feature tracking. J Cardiovasc Magn Reson.

[7] Carlsson M, Heiberg E, Ostenfeld E (2018). Functional contribution of circumferential versus longitudinal strain. J Am Coll Cardiol.

[8] Carlsson M, Ugander M, Heiberg E, Arheden H (2007). The quantitative relationship between longitudinal and radial function in left, right, and total heart pumping in humans. Am J Physiol Circ Physiol.

[9] Ostenfeld E, Stephensen SS, Steding-Ehrenborg K (2016). Regional contribution to ventricular stroke volume is affected on the left side, but not on the right in patients with pulmonary hypertension. Int J Cardiovasc Imaging.

[10] Gyllenhammar T, Kanski M, Engblom H (2018). Decreased global myocardial perfusion at adenosine stress as a potential new biomarker for microvascular disease in systemic sclerosis: a magnetic resonance study. BMC Cardiovasc Disord.

[11] Bodetoft S, Carlsson M, Arheden H, Ekelund U (2011). Effects of oxygen inhalation on cardiac output, coronary blood flow and oxygen delivery in healthy individuals, assessed with MRI. Eur J Emerg Med.

[12] Simonetti OP, Kim RJ, Fieno DS (2001). An improved MR imaging technique for the visualization of myocardial infarction. Radiology.

[13] Heiberg E, Sjögren J, Ugander M (2010). Design and validation of segment—freely available software for cardiovascular image analysis. BMC Med Imaging.

[14] Schulz-Menger J, Bluemke DA, Bremerich J (2020). Standardized image interpretation and post-processing in cardiovascular magnetic resonance—2020 update. J Cardiovasc Magn Reson.

[15] Stephensen SS, Ostenfeld E, Steding-Ehrenborg K (2018). Alterations in ventricular pumping in patients with atrial septal defect at rest, during dobutamine stress and after defect closure. Clin Physiol Funct Imaging.

[16] Seemann F, Pahlm U, Steding-ehrenborg K (2017). Time-resolved tracking of the atrioventricular plane displacement in Cardiovascular Magnetic Resonance (CMR) images. BMC Med Imaging.

[17] Carlsson M, Ugander M, Mosén H (2007). Atrioventricular plane displacement is the major contributor to left ventricular pumping in healthy adults, athletes, and patients with dilated cardiomyopathy. Am J Physiol Heart Circ Physiol.

[18] Pávek K, Boska D, Selecky FV, Sauberer A (1964). Measurement of cardiac output by thermodilution with constant rate injection of indicator. Circ Res.

[19] Baggen V, Driessen MMP, Post M (2016). Echocardiographic findings associated with mortality or transplant in patients with pulmonary arterial hypertension: a systematic review and meta-analysis. Netherlands Hear J.

[20] Schuuring MJ, Van RACMJ, Vis JC (2015). New predictors of mortality in adults with congenital heart disease and pulmonary hypertension: midterm outcome of a prospective study. Int J Cardiol.

[21] Cho I, Oh J, Chang H (2014). Tricuspid regurgitation duration correlates with cardiovascular magnetic resonance-derived right ventricular ejection fraction and predict prognosis in patients with pulmonary arterial hypertension. Eur Hear J Cardiovasc Imaging.

[22] Sepúlveda-Martínez A, Steding-Ehrenborg K, Rodríguez-López M (2021). Atrioventricular plane displacement versus mitral and tricuspid annular plane systolic excursion: a comparison between cardiac magnetic resonance and M-mode echocardiography. Clin Physiol Funct Imaging.

[23] Evaldsson AW, Lindholm A, Jumatate R (2020). Right ventricular function parameters in pulmonary hypertension: echocardiography vs. cardiac magnetic resonance. BMC Cardiovasc Disord.

[24] Santamore WP, Dell’Italia LJ (1998). Ventricular interdependence: significant left ventricular contributions to right ventricular systolic function. Prog Cardiovasc Dis.

[25] Sato T, Ambale-Venkatesh B, Lima JAC (2018). The impact of ambrisentan and tadalafil upfront combination therapy on cardiac function in scleroderma associated pulmonary arterial hypertension patients: cardiac magnetic resonance feature tracking study. Pulm Circ.

[26] Reesink HJ, Marcus JT, Tulevski II (2007). Reverse right ventricular remodeling after pulmonary endarterectomy in patients with chronic thromboembolic pulmonary hypertension: utility of magnetic resonance. J Thorac Cardiovasc Surg.

[27] Baggen VJM, Leiner T, Post MC (2016). Cardiac magnetic resonance findings predicting mortality in patients with pulmonary arterial hypertension: a systematic review and meta-analysis. Eur Radiol.

[28] Bredfelt A, Rådegran G, Hesselstrand R (2018). Increased right atrial volume measured with cardiac magnetic resonance is associated with worse clinical outcome in patients with pre-capillary pulmonary hypertension. ESC Hear Fail.

[29] Corona-villalobos CP, Kamel IR, Rastegar N (2015). Bidimensional measurements of right ventricular function for prediction of survival in patients with pulmonary hypertension: comparison of reproducibility and time of analysis with volumetric cardiac magnetic resonance imaging analysis. Pulm Circ.

[30] Romano S, Judd RM, Kim RJ (2018). Left ventricular long-axis function assessed with cardiac cine MR imaging is an independent predictor of all-cause mortality in patients with reduced ejection fraction: a multicenter study. Radiology.

[31] van Wolferen SA, Marcus JT, Boonstra A (2007). Prognostic value of right ventricular mass, volume, and function in idiopathic pulmonary arterial hypertension. Eur Heart J.

[32] Humbert M, Sitbon O, Yaïcia A (2010). Survival in incident and prevalent cohorts of patients with pulmonary arterial hypertension. Eur Respir J.

[33] Seemann F, Berg J, Solem K (2020). Quantification of left ventricular contribution to stroke work by longitudinal and radial force-length loops. J Appl Physiol.

[34] Tezuka F, Hart W, Lange PE, Nürnberg JH (1990). Muscle fiber orientation in the development and regression of right ventricular hypertrophy in pigs. Pathol Int.

[35] Rich S (2012). Right ventricular adaptation and maladaptation in chronic pulmonary arterial hypertension. Cardiol Clin.

[36] Brown SB, Raina A, Katz D (2011). Longitudinal shortening accounts for the majority of right ventricular contraction and improves after pulmonary vasodilator therapy in normal subjects and patients with pulmonary arterial hypertension. Chest.

[37] Hsia HH, Haddad F (2012). Pulmonary hypertension interdependence?. J Am Coll Cardiol.

[38] Karas MG, Kizer JR (2012). Echocardiographic assessment of the right ventricle and associated hemodynamics. Prog Cardiovasc Dis.

[39] Mouratoglou AS, Kallifatidis A, Pitsiou G (2018). Duration of interventricular septal shift toward the left ventricle is associated with poor clinical outcome in precapillary pulmonary hypertension: a cardiac magnetic resonance study. Hell J Cardiol.

[40] Shapiro S, Rn GLT, Ms MT (2012). Sex differences in the diagnosis, treatment, and outcome of patients enrolled in the registry to evaluate. Chest.

[41] Kjellström B, Nisell M, Kylhammar D (2019). Sex-specific differences and survival in patients with idiopathic pulmonary arterial hypertension 2008–2016. ERJ Open Res.

